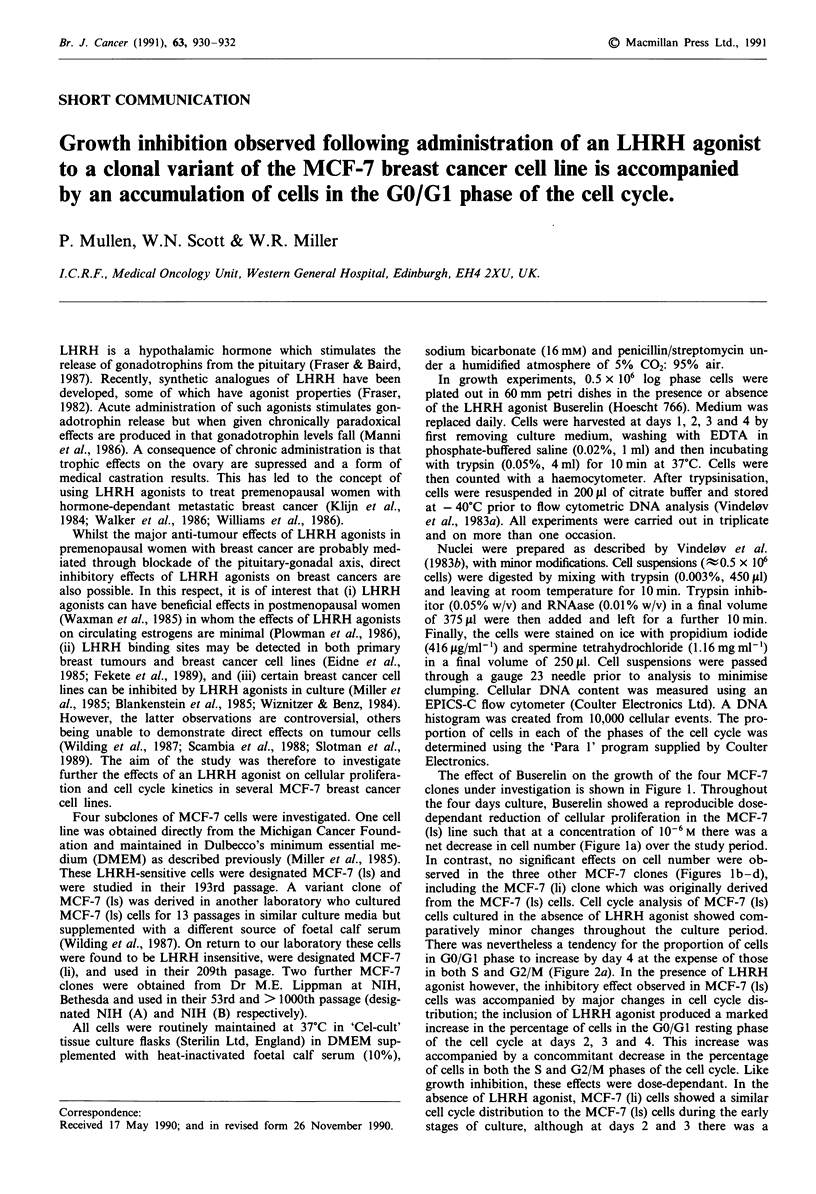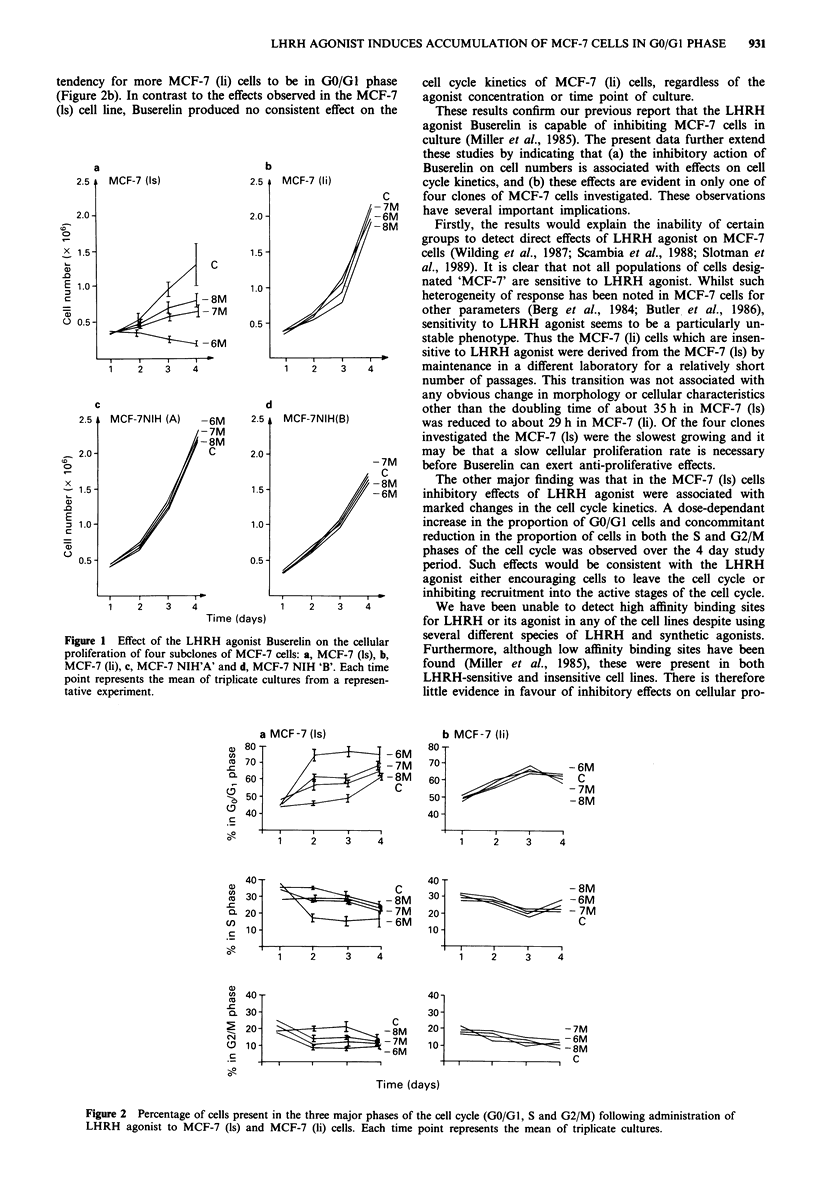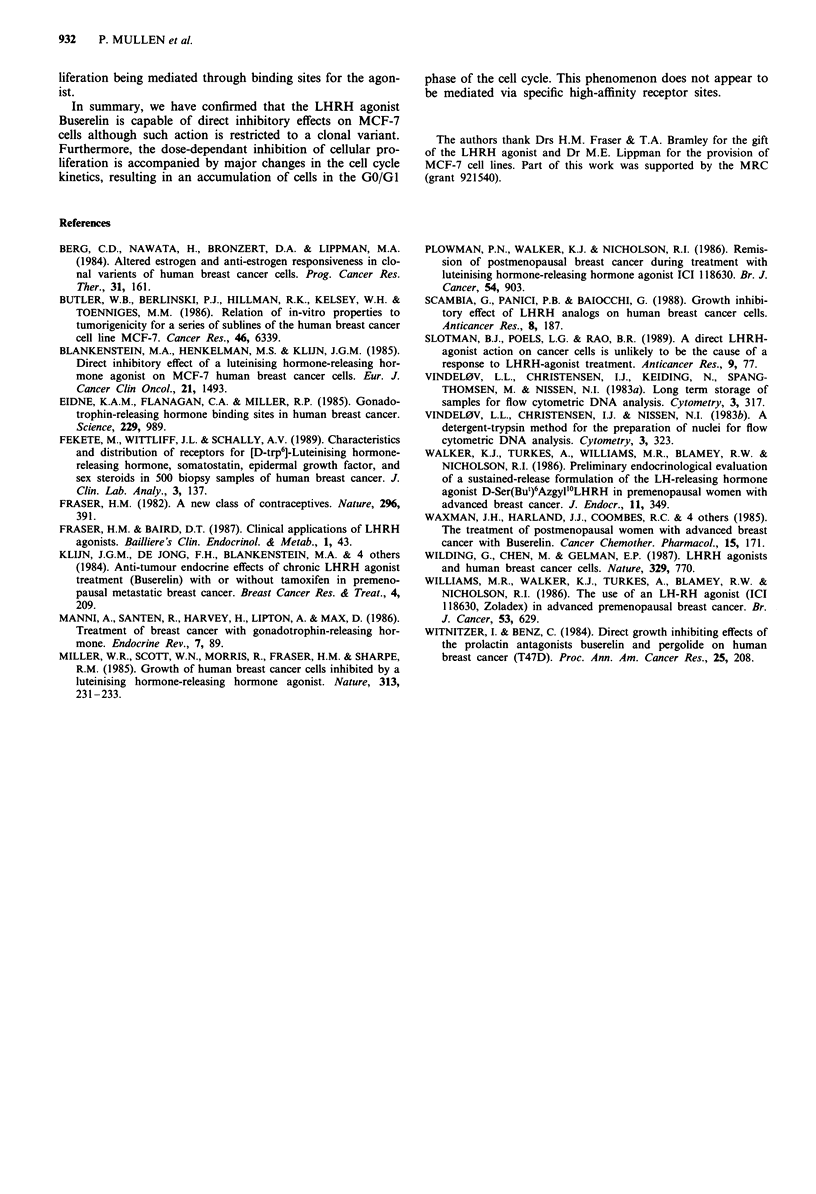# Growth inhibition observed following administration of an LHRH agonist to a clonal variant of the MCF-7 breast cancer cell line is accompanied by an accumulation of cells in the G0/G1 phase of the cell cycle.

**DOI:** 10.1038/bjc.1991.203

**Published:** 1991-06

**Authors:** P. Mullen, W. N. Scott, W. R. Miller

**Affiliations:** I.C.R.F., Medical Oncology Unit, Western General Hospital, Edinburgh, UK.


Br. J. Cancer (1991), 63, 930-932 © Macmillan Press Ltd., 1991

SHORT COMMUNICATION

### Growth inhibition observed following administration of an LHRH agonist to a clonal variant of the MCF-7 breast cancer cell line is accompanied by an accumulation of cells in the G0/G1 phase of the cell cycle.

P. Mullen, W.N. Scott & W.R. Miller

*I.C.R.F., Medical Oncology Unit, Western General Hospital, Edinburgh, EH4 2XU, UK.*

LHRH is a hypothalamic hormone which stimulates the release of gonadotrophins from the pituitary (Fraser & Baird, 1987). Recently, synthetic analogues of LHRH have been developed, some of which have agonist properties (Fraser, 1982). Acute administration of such agonists stimulates gonadotrophin release but when given chronically paradoxical effects are produced in that gonadotrophin levels fall (Manni *et al.*, 1986). A consequence of chronic administration is that trophic effects on the ovary are supressed and a form of medical castration results. This has led to the concept of using LHRH agonists to treat premenopausal women with hormone-dependant metastatic breast cancer (Klijn *et al.*, 1984; Walker *et al.*, 1986; Williams *et al.*, 1986).

Whilst the major anti-tumour effects of LHRH agonists in premenopausal women with breast cancer are probably mediated through blockade of the pituitary-gonadal axis, direct inhibitory effects of LHRH agonists on breast cancers are also possible. In this respect, it is of interest that (i) LHRH agonists can have beneficial effects in postmenopausal women (Waxman *et al.*, 1985) in whom the effects of LHRH agonists on circulating estrogens are minimal (Plowman *et al.*, 1986), (ii) LHRH binding sites may be detected in both primary breast tumours and breast cancer cell lines (Eidne *et al.*, 1985; Fekete *et al.*, 1989), and (iii) certain breast cancer cell lines can be inhibited by LHRH agonists in culture (Miller *et al.*, 1985; Blankenstein *et al.*, 1985; Wiznitzer & Benz, 1984). However, the latter observations are controversial, others being unable to demonstrate direct effects on tumour cells (Wilding *et al.*, 1987; Scambia *et al.*, 1988; Slotman *et al.*, 1989). The aim of the study was therefore to investigate further the effects of an LHRH agonist on cellular proliferation and cell cycle kinetics in several MCF-7 breast cancer cell lines.

Four subclones of MCF-7 cells were investigated. One cell line was obtained directly from the Michigan Cancer Foundation and maintained in Dulbecco's minimum essential medium (DMEM) as described previously (Miller *et al.*, 1985). These LHRH-sensitive cells were designated MCF-7 (ls) and were studied in their 193rd passage. A variant clone of MCF-7 (ls) was derived in another laboratory who cultured MCF-7 (ls) cells for 13 passages in similar culture media but supplemented with a different source of foetal calf serum (Wilding *et al.*, 1987). On return to our laboratory these cells were found to be LHRH insensitive, were designated MCF-7 (li), and used in their 209th pasage. Two further MCF-7 clones were obtained from Dr M.E. Lippman at NIH, Bethesda and used in their 53rd and > 1000th passage (designated NIH (A) and NIH (B) respectively).

All cells were routinely maintained at 37°C in 'Cel-cult' tissue culture flasks (Sterilin Ltd, England) in DMEM supplemented with heat-inactivated foetal calf serum (10%), sodium bicarbonate (16 mM) and penicillin/streptomycin under a humidified atmosphere of 5% $CO_2$: 95% air.

In growth experiments, $0.5 \times 10^6$ log phase cells were plated out in 60 mm petri dishes in the presence or absence of the LHRH agonist Buserelin (Hoescht 766). Medium was replaced daily. Cells were harvested at days 1, 2, 3 and 4 by first removing culture medium, washing with EDTA in phosphate-buffered saline (0.02%, 1 ml) and then incubating with trypsin (0.05%, 4 ml) for 10 min at 37°C. Cells were then counted with a haemocytometer. After trypsinisation, cells were resuspended in 200 µl of citrate buffer and stored at − 40°C prior to flow cytometric DNA analysis (Vindeløv *et al.*, 1983*a*). All experiments were carried out in triplicate and on more than one occasion.

Nuclei were prepared as described by Vindeløv *et al.* (1983*b*), with minor modifications. Cell suspensions ($\approx 0.5 \times 10^6$ cells) were digested by mixing with trypsin (0.003%, 450 µl) and leaving at room temperature for 10 min. Trypsin inhibitor (0.05% w/v) and RNAase (0.01% w/v) in a final volume of 375 µl were then added and left for a further 10 min. Finally, the cells were stained on ice with propidium iodide (416 $\mu g/ml^{-1}$) and spermine tetrahydrochloride (1.16 $mg\ ml^{-1}$) in a final volume of 250 µl. Cell suspensions were passed through a gauge 23 needle prior to analysis to minimise clumping. Cellular DNA content was measured using an EPICS-C flow cytometer (Coulter Electronics Ltd). A DNA histogram was created from 10,000 cellular events. The proportion of cells in each of the phases of the cell cycle was determined using the 'Para 1' program supplied by Coulter Electronics.

The effect of Buserelin on the growth of the four MCF-7 clones under investigation is shown in Figure 1. Throughout the four days culture, Buserelin showed a reproducible dose-dependant reduction of cellular proliferation in the MCF-7 (ls) line such that at a concentration of $10^{-6}$ M there was a net decrease in cell number (Figure 1a) over the study period. In contrast, no significant effects on cell number were observed in the three other MCF-7 clones (Figures 1b-d), including the MCF-7 (li) clone which was originally derived from the MCF-7 (ls) cells. Cell cycle analysis of MCF-7 (ls) cells cultured in the absence of LHRH agonist showed comparatively minor changes throughout the culture period. There was nevertheless a tendency for the proportion of cells in G0/G1 phase to increase by day 4 at the expense of those in both S and G2/M (Figure 2*a*). In the presence of LHRH agonist however, the inhibitory effect observed in MCF-7 (ls) cells was accompanied by major changes in cell cycle distribution; the inclusion of LHRH agonist produced a marked increase in the percentage of cells in the G0/G1 resting phase of the cell cycle at days 2, 3 and 4. This increase was accompanied by a concommitant decrease in the percentage of cells in both the S and G2/M phases of the cell cycle. Like growth inhibition, these effects were dose-dependant. In the absence of LHRH agonist, MCF-7 (li) cells showed a similar cell cycle distribution to the MCF-7 (ls) cells during the early stages of culture, although at days 2 and 3 there was a

Correspondence:
Received 17 May 1990; and in revised form 26 November 1990.

tendency for more MCF-7 (li) cells to be in G0/G1 phase (Figure 2b). In contrast to the effects observed in the MCF-7 (ls) cell line, Buserelin produced no consistent effect on the

**Figure 1** Effect of the LHRH agonist Buserelin on the cellular proliferation of four subclones of MCF-7 cells: **a**, MCF-7 (ls), **b**, MCF-7 (li), **c**, MCF-7 NIH'A' and **d**, MCF-7 NIH 'B'. Each time point represents the mean of triplicate cultures from a representative experiment.

cell cycle kinetics of MCF-7 (li) cells, regardless of the agonist concentration or time point of culture.

These results confirm our previous report that the LHRH agonist Buserelin is capable of inhibiting MCF-7 cells in culture (Miller *et al.*, 1985). The present data further extend these studies by indicating that (a) the inhibitory action of Buserelin on cell numbers is associated with effects on cell cycle kinetics, and (b) these effects are evident in only one of four clones of MCF-7 cells investigated. These observations have several important implications.

Firstly, the results would explain the inability of certain groups to detect direct effects of LHRH agonist on MCF-7 cells (Wilding *et al.*, 1987; Scambia *et al.*, 1988; Slotman *et al.*, 1989). It is clear that not all populations of cells designated 'MCF-7' are sensitive to LHRH agonist. Whilst such heterogeneity of response has been noted in MCF-7 cells for other parameters (Berg *et al.*, 1984; Butler *et al.*, 1986), sensitivity to LHRH agonist seems to be a particularly unstable phenotype. Thus the MCF-7 (li) cells which are insensitive to LHRH agonist were derived from the MCF-7 (ls) by maintenance in a different laboratory for a relatively short number of passages. This transition was not associated with any obvious change in morphology or cellular characteristics other than the doubling time of about 35 h in MCF-7 (ls) was reduced to about 29 h in MCF-7 (li). Of the four clones investigated the MCF-7 (ls) were the slowest growing and it may be that a slow cellular proliferation rate is necessary before Buserelin can exert anti-proliferative effects.

The other major finding was that in the MCF-7 (ls) cells inhibitory effects of LHRH agonist were associated with marked changes in the cell cycle kinetics. A dose-dependant increase in the proportion of G0/G1 cells and concommitant reduction in the proportion of cells in both the S and G2/M phases of the cell cycle was observed over the 4 day study period. Such effects would be consistent with the LHRH agonist either encouraging cells to leave the cell cycle or inhibiting recruitment into the active stages of the cell cycle.

We have been unable to detect high affinity binding sites for LHRH or its agonist in any of the cell lines despite using several different species of LHRH and synthetic agonists. Furthermore, although low affinity binding sites have been found (Miller *et al.*, 1985), these were present in both LHRH-sensitive and insensitive cell lines. There is therefore little evidence in favour of inhibitory effects on cellular pro-

**Figure 2** Percentage of cells present in the three major phases of the cell cycle (G0/G1, S and G2/M) following administration of LHRH agonist to MCF-7 (ls) and MCF-7 (li) cells. Each time point represents the mean of triplicate cultures.

liferation being mediated through binding sites for the agonist.

In summary, we have confirmed that the LHRH agonist Buserelin is capable of direct inhibitory effects on MCF-7 cells although such action is restricted to a clonal variant. Furthermore, the dose-dependant inhibition of cellular proliferation is accompanied by major changes in the cell cycle kinetics, resulting in an accumulation of cells in the G0/G1 phase of the cell cycle. This phenomenon does not appear to be mediated via specific high-affinity receptor sites.

The authors thank Drs H.M. Fraser & T.A. Bramley for the gift of the LHRH agonist and Dr M.E. Lippman for the provision of MCF-7 cell lines. Part of this work was supported by the MRC (grant 921540).

### References

BERG, C.D., NAWATA, H., BRONZERT, D.A. & LIPPMAN, M.A. (1984). Altered estrogen and anti-estrogen responsiveness in clonal varients of human breast cancer cells. *Prog. Cancer Res. Ther.*, **31**, 161.

BUTLER, W.B., BERLINSKI, P.J., HILLMAN, R.K., KELSEY, W.H. & TOENNIGES, M.M. (1986). Relation of in-vitro properties to tumorigenicity for a series of sublines of the human breast cancer cell line MCF-7. *Cancer Res.*, **46**, 6339.

BLANKENSTEIN, M.A., HENKELMAN, M.S. & KLIJN, J.G.M. (1985). Direct inhibitory effect of a luteinising hormone-releasing hormone agonist on MCF-7 human breast cancer cells. *Eur. J. Cancer Clin Oncol.*, **21**, 1493.

EIDNE, K.A.M., FLANAGAN, C.A. & MILLER, R.P. (1985). Gonadotrophin-releasing hormone binding sites in human breast cancer. *Science*, **229**, 989.

FEKETE, M., WITTLIFF, J.L. & SCHALLY, A.V. (1989). Characteristics and distribution of receptors for [D-trp$^6$]-Luteinising hormone-releasing hormone, somatostatin, epidermal growth factor, and sex steroids in 500 biopsy samples of human breast cancer. *J. Clin. Lab. Analy.*, **3**, 137.

FRASER, H.M. (1982). A new class of contraceptives. *Nature*, **296**, 391.

FRASER, H.M. & BAIRD, D.T. (1987). Clinical applications of LHRH agonists. *Bailliere's Clin. Endocrinol. & Metab.*, **1**, 43.

KLIJN, J.G.M., DE JONG, F.H., BLANKENSTEIN, M.A. & 4 others (1984). Anti-tumour endocrine effects of chronic LHRH agonist treatment (Buserelin) with or without tamoxifen in premenopausal metastatic breast cancer. *Breast Cancer Res. & Treat.*, **4**, 209.

MANNI, A., SANTEN, R., HARVEY, H., LIPTON, A. & MAX, D. (1986). Treatment of breast cancer with gonadotrophin-releasing hormone. *Endocrine Rev.*, **7**, 89.

MILLER, W.R., SCOTT, W.N., MORRIS, R., FRASER, H.M. & SHARPE, R.M. (1985). Growth of human breast cancer cells inhibited by a luteinising hormone-releasing hormone agonist. *Nature*, **313**, 231–233.

PLOWMAN, P.N., WALKER, K.J. & NICHOLSON, R.I. (1986). Remission of postmenopausal breast cancer during treatment with luteinising hormone-releasing hormone agonist ICI 118630. *Br. J. Cancer*, **54**, 903.

SCAMBIA, G., PANICI, P.B. & BAIOCCHI, G. (1988). Growth inhibitory effect of LHRH analogs on human breast cancer cells. *Anticancer Res.*, **8**, 187.

SLOTMAN, B.J., POELS, L.G. & RAO, B.R. (1989). A direct LHRH-agonist action on cancer cells is unlikely to be the cause of a response to LHRH-agonist treatment. *Anticancer Res.*, **9**, 77.

VINDELØV, L.L., CHRISTENSEN, I.J., KEIDING, N., SPANG-THOMSEN, M. & NISSEN, N.I. (1983*a*). Long term storage of samples for flow cytometric DNA analysis. *Cytometry*, **3**, 317.

VINDELØV, L.L., CHRISTENSEN, I.J. & NISSEN, N.I. (1983*b*). A detergent-trypsin method for the preparation of nuclei for flow cytometric DNA analysis. *Cytometry*, **3**, 323.

WALKER, K.J., TURKES, A., WILLIAMS, M.R., BLAMEY, R.W. & NICHOLSON, R.I. (1986). Preliminary endocrinological evaluation of a sustained-release formulation of the LH-releasing hormone agonist D-Ser(Bu$^t$)$^6$Azgyl$^{10}$LHRH in premenopausal women with advanced breast cancer. *J. Endocr.*, **11**, 349.

WAXMAN, J.H., HARLAND, J.J., COOMBES, R.C. & 4 others (1985). The treatment of postmenopausal women with advanced breast cancer with Buserelin. *Cancer Chemother. Pharmacol.*, **15**, 171.

WILDING, G., CHEN, M. & GELMAN, E.P. (1987). LHRH agonists and human breast cancer cells. *Nature*, **329**, 770.

WILLIAMS, M.R., WALKER, K.J., TURKES, A., BLAMEY, R.W. & NICHOLSON, R.I. (1986). The use of an LH-RH agonist (ICI 118630, Zoladex) in advanced premenopausal breast cancer. *Br. J. Cancer*, **53**, 629.

WITNITZER, I. & BENZ, C. (1984). Direct growth inhibiting effects of the prolactin antagonists buserelin and pergolide on human breast cancer (T47D). *Proc. Ann. Am. Cancer Res.*, **25**, 208.